# Internal consistency and measurement equivalence of the cannabis screening questions on the paper-and-pencil face-to-face ASSIST versus the online instrument

**DOI:** 10.1186/s13011-015-0002-9

**Published:** 2015-03-08

**Authors:** Yasser Khazaal, Anne Chatton, Grégoire Monney, Audrey Nallet, Riaz Khan, Daniele Zullino, Jean-François Etter

**Affiliations:** Geneva University Hospitals, Grand pré, 70 C 1202, Geneva, Switzerland; Geneva University, Geneva, Switzerland; Institute of Global Health, Faculty of Medicine, University of Geneva, Geneva, Switzerland

**Keywords:** The alcohol, Smoking and substance involvement screening test, Cannabis, Psychometrics, Screening test, Internet

## Abstract

**Background:**

Validated Internet-based screening tools for cannabis use and abuse are needed. The present study aimed to establish equivalence between the previously validated Alcohol, Smoking and Substance Involvement Screening Test (ASSIST) as a paper-and-pencil (PaP)-administered questionnaire and its online use.

**Methods:**

Two groups of cannabis users took part in this study and the results were analyzed using structural equation modeling. One group consisted of 150 participants and was assessed with the ASSIST PaP questionnaire in a face-to-face interview (the PaP group). They were recruited from three settings: a primary health care outpatient clinic, a general psychiatric facility, and an ambulatory specialized addiction treatment facility. The other group (the Web group) comprised 1382 persons who answered the online version of the same questionnaire. This sample was drawn from people who naturalistically visited a website dedicated to helping people with cannabis addiction.

**Results:**

The internal consistency was good for the online questionnaire (0.74) and high for the already validated PaP questionnaire (0.91). The Web group, however, had higher scores on cannabis use than did the PaP group. The results show support for configural invariance, meaning that the one-factor structure was preserved across groups, although measurement equivalence between these two survey modes was not achieved. However, when the Web group was split into two random subsamples, measurement invariance was demonstrated between them by cross-validation.

**Conclusions:**

Measurement equivalence was not achieved between the two survey modes. Nonetheless, subanalyses of the Web group demonstrated that the cannabis screening questions of the ASSIST can be used for online screening. Differences in ASSIST scores between samples may be due to the sensitive nature of the information surveyed, with possible underreporting in face-to-face interviews, or to the different characteristics of the Web group because of the specialized nature of the website.

## Background

The Internet has come to be widely used as a tool for drug treatment and research. For instance, the Web is now commonly used in searches for medical and psychological information [1-3]. Furthermore, different forms of online treatments have been studied for various mental health and addiction-related conditions [4-6]. In consequence, an increased number of medical and psychological studies have been performed by using Web-based questionnaires [7], including topics related to substance use [8].

Internet-based surveys have a number of advantages in comparison with paper-and-pencil (PaP) questionnaires [7,9-11], such as easy data collection and a reduction in data entry errors. The wide dissemination of online surveys may also lead to an increase in recruitment and lower desirability bias compared with traditional methods of data collection. The latter is of particular interest for surveys on stigmatizing topics such as drug use [7]. For example, Web-based, self-administered questionnaires lead to higher reporting rates of substance use in adolescents compared with the rates reported from PaP self-administered questionnaires [12]. Furthermore, the quality of the data provided by Internet-based surveys on topics such as smoking [13] and alcohol use [14] have equal or even better reliability in Web-based questionnaires than in PaP or face-to-face approaches. The Internet, probably because of the perception that it offers anonymity, has also been found to be a useful tool in reaching stigmatized populations, such as illicit drug users and those who use party drugs such as ecstasy or cannabis [15]. The feasibility of using online research for substance use and illegal substance use has been demonstrated in several studies [15].

Despite the wide dissemination of Internet-based surveys, participants in such studies may differ from the general population. In particular, they may be more educated or more likely to be employed [16,17]. Nevertheless, if the possible limitations are acknowledged, Internet-based surveys are suitable for research on topics related to substance use and addictive behaviors, in particular to generate hypotheses that can later be tested in more representative samples [15].

Regardless of the number of opportunities linked to online research, however, some concerns remain regarding the reliability (whereby a given procedure produces the same results in tests repeated with the same empirical or equivalent tools) [18] and validity of the data (the degree to which a given procedure operationalizes the intended concept) [18] obtained via online questionnaires [7]. Studies investigating the invariance of Web-based questionnaires and PaP surveys have demonstrated inconsistent findings. Some studies found no major differences between the two delivery modes [19], whereas others found variations related to particular scales [20] and still others reported differences between online and offline versions of the same tests in terms of score distributions achieved and psychometric properties of the tests [20,21]. The latter findings are particularly true for studies related to self-disclosure of sensitive information [22]. Social desirability may affect answers in face-to-face interviews [23]. For instance, reports of alcohol use and sexually risky behaviors were more likely to be fully disclosed in Web surveys than in traditional formats [24]. Also noteworthy is the possible impact of different designs on the outcomes in these studies. Some researchers used within-subjects designs, whereas others used between-subjects designs in which the online questionnaire or the PaP form was randomly administered, or not administered, to the participants [25].

While an increased number of instruments classically used for mental health assessments have been developed and validated for Internet use [26-29], such developments are needed for the study of addictive disorders, especially for frequently used substances such as cannabis [30-33]. In the last few years, international efforts have been made to develop and validate screening assessment tools related to cannabis use [34]. In particular, after the success of the Alcohol Use Disorder Identification Test (AUDIT) [35], efforts were made to offer similar screening tools for other substance use, including cannabis. The Alcohol, Smoking and Substance Involvement Screening Test (ASSIST) [36,37] was developed by the World Health Organization to screen for problematic or risky substance use (http://www.who.int/substance_abuse/activities/assist/en/index.html). The ASSIST instrument was validated as a face-to-face questionnaire in different populations and in a number of linguistic versions, in which it was found to have sound psychometric properties. Its internal consistency, assessed with Cronbach’s α, ranged from 0.74 to 0.93. Concurrent validity was demonstrated by significant correlations between ASSIST scores and scores from the Addiction Severity Index [38], AUDIT [35], Revised Fagerstrom Tolerance Questionnaire [39], and Mini International Neuropsychiatric Interview (MINI-Plus) [40] for the diagnosis of substance abuse or dependence. Moreover, the ASSIST yielded interesting sensitivity and specificity values for discrimination between substance use and abuse and between substance abuse and dependence [37,41-44]. In its current version (V3.0), the ASSIST comprises eight questions for each of the following substances: tobacco, alcohol, cannabis, cocaine, amphetamine-type stimulants, inhalants, sedatives, hallucinogens, opiates, and other drugs. Question 1 deals with the lifetime use of each substance. If a substance is used, people are invited to answer additional questions related to this specific substance.

PaP and Web-based surveys may not produce similar results; therefore, measurement equivalence of Web-based and PaP surveys cannot be taken for granted and must be empirically demonstrated [45,46]. To our knowledge, the ASSIST, despite the possibility of its use in naturalistic online settings, has not yet been used in Internet studies and has yet to be validated for online studies.

In the current study, we aimed to do the following:

1. Describe the distribution of self-reported cannabis use in two samples of cannabis users: visitors to a cannabis prevention website who answered an online questionnaire, and patients of three clinics who answered a PaP questionnaire in a face-to-face interview. Both groups answered the ASSIST version 3.0 questionnaire.
2. Analyze whether the items in the ASSIST instrument operate equivalently across Internet and PaP groups for gathering sensitive information (i.e., is the measurement model group invariant?).

## Methods

### Participants and procedures

We obtained two groups of cannabis users. The first group was assessed with the ASSIST PaP questionnaire on a face-to-face basis, and the second group answered an online version of the same questionnaire.

### The first group

The first group consisted of the adults who took part in a previous study related to the validation of the French version of the ASSIST questionnaire [44]. It included 150 participants, of whom 50 were patients from a primary health care outpatient clinic, 50 were outpatients from a general psychiatric facility, and 50 were patients from an ambulatory specialized addiction treatment facility. The use of tobacco, alcohol, and illicit substances (cannabis, cocaine, opiates, etc.) was assessed. The three settings were concerned with these substances, except for the primary care setting, where cocaine and opiates were not identified as problematic. Opiate dependence was found in 7.3% of the sample, alcohol abuse or dependence in 43.3%, and cannabis use in 85.3%. Of the latter, 6.3% were considered to have cannabis abuse or dependence. The mean age of the participants was 41 ± 11.5 years. They were mainly men (64%), single (76.7%), and not working (68.5%). All participants treated in these settings were eligible if they were older than 18 years, able to speak in French, and able to give their written informed consent.

The ASSIST (PaP) assessment (in French) was made during an interview with a trained psychologist or psychiatrist who completed the ASSIST during the interview. The ethics committee of the Geneva University Hospitals approved the study.

### The second group

The second group was drawn from people who naturalistically visited (without specific advertisement or invitation) a French-language website dedicated to online cannabis help (stop-cannabis.ch). The website offers information and online help for cannabis users. In particular, visitors may access a number of automated interventions such as an online motivational interview, a brief intervention (computer-tailored feedback based on the ASSIST scores), and support for cannabis cessation in a text-messaging format. Although the website is primarily dedicated to people asking for information or help related to cannabis use, it is accessible to anyone, and no specific registration is required to use it. It also includes information for the relatives of cannabis users. The website has been visited by 676,000 Internet users since 2008. No special advertising strategies promote the website: It is mostly accessed via keyword search on general search engines such as Google, as well as from links on other websites such as a sister website dedicated to tobacco smokers. Participants could answer the ASSIST questions related to cannabis use online without any registration procedure and without answering additional socio-demographic questions. After answering the questionnaire (in French), participants received, on a new Web page, a personalized, computer-tailored feedback report based on their answers (one graph and 250 words) that commented on their cannabis use and recommended treatment if necessary. We did not collect any demographic information about participants. Answers emerging from the same IP address were excluded. Participants had the option to accept or decline storage of their answers for survey purposes.

### Measurements

After a positive screening result on lifetime substance use of cannabis (ASSIST, Question1), both groups of participants answered the following questions:

Questions 2 to 7 of the ASSIST:

Q2: In the past three months how often have you used cannabis?
Q3: During the past three months how often have you had a strong desire or urge to use cannabis?
Q4: During the past three months how often has your use of cannabis led to health, social, legal or financial problems?
Q5: During the past three months how often have you failed to do what was normally expected of you because of your use of cannabis?
Q6: Has a friend or relative or anyone else ever expressed concern about your use of cannabis?
Q7: Have you ever tried to control, cut down or stop using cannabis?

Questions 2 to 5 are rated on a 5-point Likert scale ranging from “never” (in the past 3 months) to “daily or almost daily”, whereas Questions 6 and 7 use a three-category rating (“no never”, “yes in the past 3 months”, “yes but not in the past 3 months”). The total score, calculated as the sum of scores received for Questions 2 through 7 inclusive, is bound to measure one latent factor: the specific substance involvement score as defined by the ASSIST, here the cannabis involvement score.

This score enabled us to identify three groups of cannabis users:

- Low risk (score: 0–3): The participant is at low risk of health and other problems from his/her current pattern of use.
- Moderate risk (score: 4–26): The participant is at risk of health and other problems from his/her current pattern of substance use.
- High risk (score ≥ 27): The participant is at high risk of experiencing severe problems (health, social, financial, legal, relationship) as a result of his/her current pattern of use and is likely to be dependent.

### Statistical analyses

SPSS 18.0 (Statistical Package for the Social Sciences, SPSS Inc., Chicago, IL) and AMOS 19.0 (Analysis of Moment Structures; SPSS Inc., Chicago, IL) software programs were used to perform the statistical analyses. The distribution of self-reported cannabis use was compared by type of survey using *χ*^2^ tests.

We next assessed internal consistency of the subscale in the Web survey by Cronbach's alpha coefficient. Ideally, α should be above 0.70, but not much higher than 0.90 [47].

For the multigroup invariance hypothesis, we used the structural equation modeling (SEM) procedure described by Jöreskog [48]. Depending on the research question, searching for group equivalence may imply a series of tests performed in the following restrictive order: configural equivalence, measurement equivalence, and structural equivalence. *Configural invariance* testing focused on the extent to which the number of factors and the pattern of their structure are similar between groups. In testing for *measurement and structural invariance*, we focused more specifically on the extent to which parameters in the measurement and structural components of the model are equivalent across groups [49-51]. It is worth noting, however, that the determination of an appropriate baseline model is required for each group separately, from which the configural model is derived. Given that one of our research questions concerns measurement equivalence across groups, statistical analyses focus on configural invariance and measurement invariance.

### Evaluation of model fit

A model’s goodness of fit is examined through various indices, as described below [52-54]:

- The *χ*^2^ to degrees of freedom ratio (*χ*^2^/df)
- The comparative fit index (CFI)
- The root mean square error of approximation (RMSEA)

Changes in goodness-of-fit statistics were also examined to detect differences in the models. A significant difference in *χ*^2^ values between nested models means that all equality constraints do not hold across the groups. Since the variables are measured on an ordinal scale with relatively few categories and the values between categories are not equidistant, asymptotically distribution-free estimation instead of maximum likelihood estimation was used here as one of the strategies to accommodate non-normally distributed data.

### Sample size considerations

Sample size plays an important role in providing unbiased parameter estimates and accurate model fit information. Bentler and Chou [55] recommended a ratio of at least 5:1 of subjects to variables for normal and elliptical distributions, and there seems to be a general consensus among researchers to adopt this ratio. However, for categorical or non-normally distributed variables, as is the case here, larger samples are required than for continuous or normally distributed variables. A ratio of at least 10 subjects per variable for this type of distribution is recommended [55,56]. The sample in the current study fulfills this requirement.

## Results

A sample of 1382 persons participated in the Web study, whereas 150 persons filled in the PaP questionnaire. In the Web survey, 1% of the participants were excluded because of missing data, leaving a sample size of 1366 for the analysis. There were no missing data in the PaP survey.

### Cannabis use

There were statistically significant differences between the two groups for all ASSIST questions (Table 1). Participants from the Web survey scored higher on all items.

**Table 1:** **Self-reported cannabis use by group and questionnaire format (expressed in %), using Questions 2 to 7 of the ASSIST V3.0 questionnaire**

**Question**	**Format**		***χ*** ^**2**^	**p**
	**Outpatients PaP (n = 150)**	**Web (n = 1366)**		
2. In the past three months how often have you used cannabis?			830.2	<0.0005
- Never	75.3	2.5		
- One or two times	3.3	3.9		
- Monthly	5.3	4.2		
- Weekly	6.0	15.4		
- Daily or almost daily	10.0	74.0		
3. During the past three months how often have you had a strong desire or urge to use cannabis?			381. 9	<0.0005
- Never	78.0	12.3		
- One or two times	4.0	14.0		
- Monthly	2.7	5.3		
- Weekly	6.7	18.4		
- Daily or almost daily	8.7	50.1		
4. During the past three months how often has your use of cannabis led to health, social, legal or financial problems?			110.9	<0.0005
- Never	88.6	45.0		
- One or two times	3.4	21.2		
- Monthly	2.0	13.3		
- Weekly	2.7	11.8		
- Daily or almost daily	3.4	8.7		
5. During the past three months how often have you failed to do what was normally expected of you because of your use of cannabis?			168.7	<0.0005
- Never	96.0	40.5		
- One or two times	0	22.3		
- Monthly	0.7	10.9		
- Weekly	2.7	15.6		
- Daily or almost daily	0.7	10.8		
6. Has a friend or relative or anyone else ever expressed concern about your use of cannabis?			147. 8	<0.0005
- No, never	60.2	35.3		
- Yes, in the past three months	9.3	17.2		
- Yes, but not in the past three months	6.0	47.5		
7. Have you ever tried to control, cut down or stop using cannabis?			157.7	<0.0005
- No, never	88.7	35.3		
- Yes, in the past three months	5.3	25.8		
- Yes, but not in the past three months	6.0	38.9		
Cannabis involvement score (sum of responses to Q2-Q7)	4.2 (8.7)	22.8 (9.9)	−24.3*	<0.0005
Risk level according to ASSIST v3.0			874.3	<0.0005
- Low (0–3)	77.3	3.3		
- Moderate (4–26)	18.7	54.2		
- High (≥27)	4.0	42.5		

*Obtained by *t*-test.

### Internal consistency

According to the validation study of the French version of the ASSIST, the internal consistency of the cannabis involvement scale yielded a Cronbach’s alpha coefficient of 0.91 [44]. In the current study, these coefficients were 0.91 and 0.74 for the PaP and the Web surveys, respectively.

### Testing for configural equivalence

As noted earlier, a prerequisite to testing for instrument equivalence is to establish a well-fitting model for each group separately, followed by a combined baseline model in which the same parameters are estimated again within the framework of a multigroup model. Results showed no misspecification for the PaP model, whereas possible misspecifications regarding two error terms (Items 2 and 3, 3 and 7) were highlighted for the Web model. It was subsequently respecified and reestimated with these two error covariances included. These baseline models were considered optimal in representing the data for the PaP sample (*χ*^2^/df = 1.93; CFI = 0.90; RMSEA = 0.08) and for the Web sample (*χ*^2^/df = 1.97; CFI = 0.99; RMSEA = 0.03).

Next, these differentially specified baseline models were incorporated into one file for the purposes of testing cross-group equivalence. Assessment of this model revealed a good fit to the data, as indicated by the CFI (0.99) and RMSEA (0.02). The *χ*^2^ value (8.06, df *=* 6) provides the baseline value against which all subsequent tests for invariance are compared (Table 2).

**Table 2:** Summary of goodness-of-fit statistics for tests of invariance across format types (PaP and web format)

**Model description**	**RMSEA**	**CFI**	***χ*** ^**2**^	**df**	**Δχ** ^**2**^	**Δdf**	**Statistical significance**
1. Configural model: no constraints imposed	0.02	0.99	8.06	6	-	-	-
2. Factor loadings: constrained equally across groups	0.03	0.97	45.44	19	37.38	13	p < 0.0005

### Testing for measurement equivalence

A model with loadings constrained to be equal across groups had a fit that was significantly poorer than that of the unconstrained model (CFI = 0.97 and RMSEA = 0.03). As can be seen from Table 2, the *χ*^2^ difference value between the constrained model and the configural model is statistically significant. This finding suggests that the constrained model is significantly worse than the unconstrained model and argues for nonequivalence of factor loadings across the groups. In other words, the hypothesis that the PaP survey and the Web survey have the same factor means is rejected. The factor loadings by type of format are displayed in Table 3.

**Table 3:** **Factor loadings by format type**

**Question**	**Pen-and-paper (n = 150)**	**Web (n = 1366)**
Q2: In the past three months how often have you used cannabis?	0.92	**0.51**
Q3: During the past three months how often have you had a strong desire or urge to use cannabis?	0.98	**0.62**
Q4: During the past three months how often has your use of cannabis led to health, social, legal or financial problems?	0.76	**0.71**
Q5: During the past three months how often have you failed to do what was normally expected of you because of your use of cannabis?	0.24	**0.67**
Q6: Has a friend or relative or anyone else ever expressed concern about your use of cannabis?	0.46	**0.44**
Q7: Have you ever tried to control, cut down or stop using cannabis?	0.22	**0.50**

### Testing for invariance across two random subsamples of the Web sample

From the results reported above, the equivalence of the online and the offline versions of the ASSIST has not been demonstrated. To check whether the ASSIST could be used in online studies, we randomly split the Web sample into two comparable subgroups from the perspective of the ASSIST risk level (Table 4). We then applied the SEM methodology described earlier to the first subsample and cross-validated the results by using the other subsample in a multigroup analysis.

**Table 4:** **Self-reported cannabis use by random subsample (expressed in %), using the cannabis-related questions of the ASSIST V3.0 questionnaire**

**Question**	**Web total sample (n = 1366)**	**Web subsample 1 n = 675**	**Web subsample 2 n = 691**	**p** ^**1**^
2. In the past three months how often have you used cannabis?				1.0
- Never	2.5	2.5	2.5	
- One or two times	3.9	3.9	3.9	
- Monthly	4.2	4.1	4.3	
- Weekly	15.4	16.0	14.8	
- Daily or almost daily	74.0	73.5	74.5	
3. During the past three months how often have you had a strong desire or urge to use cannabis?				0.6
- Never	12.3	12.6	12.2	
- One or two times	14.0	15.3	12.6	
- Monthly	5.3	5.3	4.9	
- Weekly	18.4	17.9	18.8	
- Daily or almost daily	50.1	48.9	51.5	
4. During the past three months how often has your use of cannabis led to health, social, legal or financial problems?				0.8
- Never	45.0	44.4	45.7	
- One or two times	21.2	22.4	19.8	
- Monthly	13.3	13.2	13.5	
- Weekly	11.8	11.3	12.3	
- Daily or almost daily	8.7	8.7	8.7	
5. During the past three months how often have you failed to do what was normally expected of you because of your use of cannabis?				0.8
- Never	40.5	41.5	39.4	
- One or two times	22.3	21.0	23.7	
- Monthly	10.9	10.8	11.1	
- Weekly	15.6	15.7	15.2	
- Daily or almost daily	10.8	11.0	10.6	
6. Has a friend or relative or anyone else ever expressed concern about your use of cannabis?				0.3
- No, never	35.3	34.2	36.0	
- Yes, in the past three months	17.2	16.1	18.2	
- Yes, but not in the past three months	47.5	49.6	45.7	
7. Have you ever tried to control, cut down or stop using cannabis?				0.7
- No, never	35.3	36.0	35.2	
- Yes, in the past three months	25.8	24.7	26.8	
- Yes, but not in the past three months	38.9	39.3	38.1	
Cannabis involvement score (sum of responses to Q2-Q7)	22.8 (9.9)	22.8 (9.8)	22.8 (10.0)	1.0
Risk level according to ASSIST v3.0				0.9
- Low (0–3)	3.3	3.1	3.6	
- Moderate (4–26)	54.2	53.8	53.8	
- High (≥27)	42.5	43.1	42.5	

p-Values resulting from comparison of Subsample 1 and Subsample 2.

The baseline results showed good model fit for Subsample 1 (*χ*^2^/df = 1.77; CFI = 0.99; RMSEA = 0.03), as well as for Subsample 2 (*χ*^2^/df = 2.06; CFI = 0.99; RMSEA = 0.04). The next step consisted in establishing configural invariance for both groups together without constraint: it also yielded satisfactory results. As can be seen in Table 5, configural invariance (Model 1) is supported, indicating that the one-factor structure in each group is preserved. From Model 2 to Model 4, all successive invariance tests (metric invariance, residual variance invariance, strict factorial invariance) held, given the nonsignificant difference in the *χ*^2^ values (Table 5).

**Table 5:** **Summary of goodness-of-fit statistics for invariance tests across the two random subsamples**

**Model description**	**RMSEA**	**CFI**	***χ*** ^**2**^	**df**	***χ*** ^**2**^ **/df**	**Δχ** ^**2**^	**Δdf**	**Statistical significance**
**1. Configural invariance: no constraints imposed**	**0.03**	**0.99**	**26.80**	**14**	**1.9**	**-**	**-**	**-**
**2. Factor loadings: constrained equally across groups**	**0.02**	**0.99**	**32.31**	**19**	**1.7**	**5.51**	**5**	**p = 0.4**
**3. Factor loadings and variances: constrained equally**	**0.02**	**0.99**	**32.33**	**20**	**1.6**	**5.53**	**6**	**p = 0.5**
**4. Factor loadings, variances, and error covariances: constrained equally**	**0.02**	**0.99**	**38.76**	**28**	**1.4**	**11.97**	**14**	**p = 0.6**

## Discussion

In this study, the internal consistency and the validity of the ASSIST (for the assessment of cannabis use) were compared in two samples: visitors of a cannabis prevention website who answered an online survey in French, and outpatients of three clinics in Switzerland who answered an ASSIST PaP questionnaire. The internal consistency was high for the PaP survey and good for the Web survey (0.91 and 0.74, respectively). A possible explanation for this difference in value is that Cronbach’s alpha is sensitive to deviations from normality. Therefore, if the data do not meet the assumptions of normality and linearity, as in the Web sample, the reliability may be underestimated by the current formulas used for the calculation of this value [57]. Regarding validity, the results show that the study supports configural invariance, meaning that the one-factor structure is preserved across groups.

Measurement invariance was not supported by the study results, however. Cannabis use was more frequent and problematic for the Web group than it was for the clinic-recruited sample. Only 10% of the PaP sample reported daily use of cannabis in the past 3 months, in comparison to 74% of the Web sample (Question 2). As shown in Table 1, very few PaP participants (4%) admitted to having failed in tasks normally expected from them because of their cannabis use (Question 4), whereas 60% of the Web sample endorsed this item. Consequently, the loading of this item in the factor analysis (Table 3) was very different across groups. The same problem applies to Question 7 (tried to stop or cut down).

These results may be explained, in part, by the absence of a randomization procedure (on the PaP and total Web sample), leading to selection bias, one of the limitations of the study [11,58]. The characteristics of the Web sample (from the naturalistic visitors of a website dedicated to helping people facing cannabis addiction) are probably explained by the involvement (via self-selection) of participants who were more concerned by their cannabis use than would be the case in the general population, as suggested by another study on the self-selection bias associated with online studies [59]. The participants from the stop-cannabis.ch website selected themselves to visit the website and chose to assess their cannabis use with the ASSIST, probably in relation to concerns about their cannabis consumption. The participants from the clinic centers, in contrast, may not have been preoccupied by their cannabis use [44].

The contribution of a desirability bias to the differences observed between the PaP and the Web group should also not be ignored. This bias was previously reported in face-to-face interviews [7]. The phenomenon leads to underreporting of stigmatized behaviors (i.e. cannabis use) in face-to-face settings in comparison to that in computerized self-assessments [60,61]. Furthermore, differences in the understanding of some questions cannot be fully ruled out [7,58].

### Study limitations and strengths

This study has several limitations, the most important of which is that we did not include randomly equivalent groups by type of survey. This limitation may have undermined external validity. It further highlights selection [59] and desirability biases [45,62], hence restricting the statistical comparison of the PaP and the Web groups to a descriptive level. Another limitation is a lack of other clinical assessments. Comparison with a diagnostic instrument, for instance, would allow us to calculate the sensitivity and the specificity of the Internet-administered ASSIST, two inextricably linked measures that help clinicians in deciding whether to rule a diagnosis in or out. A third limitation is that detailed socio-demographic characteristics of the Internet sample were not available to examine the profile of the respondents.

These limitations having been acknowledged, the study nonetheless has two major strengths. The first is that, owing to the large Web-sample size, it has been possible to analyze two randomly selected groups whose measurement equivalence results support the view that the ASSIST is a valid instrument in Web surveys.

The second strength that must be highlighted is that, although some studies have been performed on the online assessment of substance use [63] or on Internet-based prevention [64] or treatment [65,66] of cannabis use, our study is, to the best of our knowledge, the first to describe cannabis use of naturalistically self-selected users of a specialized website. This is an important addition, demonstrating the acceptability of such websites in naturalistic settings by cannabis users who are concerned with their behavior.

## Conclusions

Despite the limitations, it appears that the cannabis screening questions of the ASSIST instrument are useful in a Web-based format. The instrument could be used for screening and then possibly for online prevention and intervention. Further studies using the ASSIST for the assessment of other addictions, in other populations, or under different conditions while using the same statistical designs (e.g. random within-subjects, between-subjects designs) are warranted to generalize these findings.
